# Association between C-Reactive Protein Velocity and Left Ventricular Function in Patients with ST-Elevated Myocardial Infarction

**DOI:** 10.3390/jcm11020401

**Published:** 2022-01-13

**Authors:** Ariel Banai, Dana Levit, Samuel Morgan, Itamar Loewenstein, Ilan Merdler, Aviram Hochstadt, Yishay Szekely, Yan Topilsky, Shmuel Banai, Yacov Shacham

**Affiliations:** Department of Cardiology, Tel-Aviv Sourasky Medical Center, Sackler Faculty of Medicine, Tel-Aviv University, Tel Aviv 64239, Israel; arielbanai@gmail.com (A.B.); levit.dana@gmail.com (D.L.); samuelmorgan@mail.tau.ac.il (S.M.); italoewe@me.com (I.L.); ilanmerdler@gmail.com (I.M.); aviramho@gmail.com (A.H.); yishays@tlvmc.gov.il (Y.S.); yant@tlvmc.gov.il (Y.T.); shmuelb@tlvmc.gov.il (S.B.)

**Keywords:** STEMI, CRP velocity, systolic dysfunction, diastolic dysfunction

## Abstract

C-reactive protein velocity (CRPv), defined as the change in wide-range CRP concentration divided by time, is an inflammatory biomarker associated with increased morbidity and mortality in patients with ST elevation myocardial infarction (STEMI) treated with primary percutaneous intervention (PCI). However, data regarding CRPv association with echocardiographic parameters assessing left ventricular systolic and diastolic function is lacking. Echocardiographic parameters and CRPv values were analyzed using a cohort of 1059 patients admitted with STEMI and treated with primary PCI. Patients were stratified into tertiles according to their CRPv. A receiver operating characteristic (ROC) curve was used to evaluate CRPv optimal cut-off values for the prediction of severe systolic and diastolic dysfunction. Patients with high CRPv tertiles had lower left ventricular ejection fraction (LVEF) (49% vs. 46% vs. 41%, respectively; *p* < 0.001). CRPv was found to independently predict LVEF ≤ 35% (HR 1.3 CI 95% 1.21–1.4; *p* < 0.001) and grade III diastolic dysfunction (HR 1.16 CI 95% 11.02–1.31; *p* = 0.02). CRPv exhibited a better diagnostic profile for severe systolic dysfunction as compared to CRP (area under the curve 0.734 ± 0.02 vs. 0.608 ± 0.02). In conclusion, For STEMI patients treated with primary PCI, CRPv is a marker of both systolic and diastolic dysfunction. Further larger studies are needed to support this finding.

## 1. Introduction

In acute ST-elevation myocardial infarction (STEMI), ischemic injury and myocardial necrosis incite an inflammatory reaction resulting in the release of various markers. One of the most widely researched inflammatory markers is C-reactive Protein (CRP). An elevation in CRP levels has an established association with adverse clinical outcomes as well as mortality in patients presenting with STEMI [1,2,3]. While CRP has been proven to be a useful biomarker for predicting many adverse outcomes in STEMI patients, the CRP level changes over time—CRP velocity (CRPv), may be a more sensitive biomarker for identifying patients’ inflammatory state and risk of developing subsequent heart failure. Recent research demonstrated that increased CRPv is associated with short-term complications in patients presenting with acute myocardial infarction [4,5], however, little is known about its possible association to left ventricular systolic and diastolic function. Past studies proved that an elevation in CRP levels is associated with a reduced diastolic left ventricular (LV) function as well as systolic heart failure in the setting of STEMI [1,6]. As CRPv is a more sensitive biomarker, it might improve the detection of patients with STEMI that are at increased risk of developing complications associated with systolic and diastolic dysfunction. The aim of this study was to evaluate the association between CRPv with LV systolic and diastolic function in patients presenting with STEMI and treated with primary percutaneous angiography intervention (PCI).

## 2. Materials and Methods

**Figure 1:**
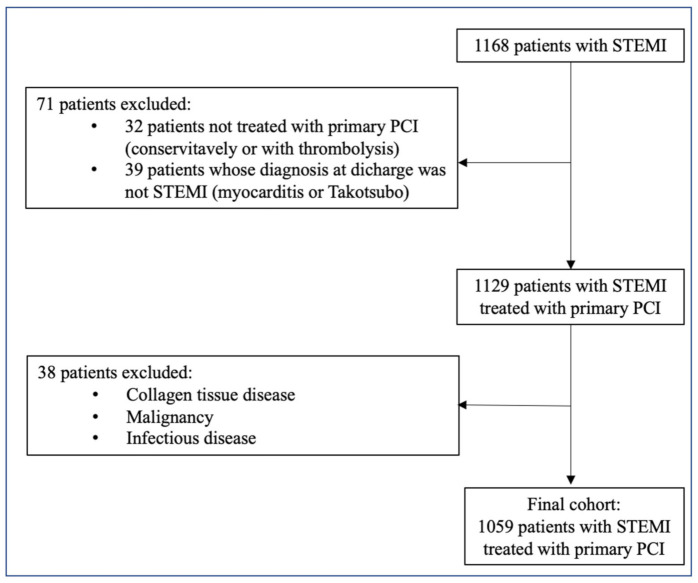
Inclusion and exclusion criteria. PCI—Percutaneous coronary intervention, STEMI—ST elevation myocardial infection.

We performed a retrospective, single-center observational study at the Tel Aviv Sourasky Medical Center, a tertiary referral hospital with a 24/7 primary PCI service. Included in the study were 1168 patients admitted to the Cardiac Intensive Care Unit (CICU) for an acute STEMI between June 2012 and November 2019. Each patient had a minimum of two successive, wide-range-CRP (wr-CRP) level measurements taken within the first 24 h of hospital admission. We excluded 32 patients who were treated either conservatively or with thrombolysis and 39 patients whose final diagnosis on discharge was other than STEMI (e.g., myocarditis or Takotsubo cardiomyopathy). An additional 38 patients were excluded because of known collagen tissue disease, advanced liver disease, malignancy, or any known infectious disease. A clear diagnosis of infection was assumed for patients with a clinically suspected infection and a positive blood culture, positive urine culture, or a consolidation on chest radiography with or without a positive sputum culture. The final population included 1059 patients (Figure 1), whose baseline demographic, cardiovascular history, clinical risk factors, treatment characteristics, and laboratory results were retrieved from their medical files. For patients who died within 30 days of admission, we surveyed all potential events that may have influenced the change in wr-CRP concentration. 

The diagnosis of STEMI was obtained by a history of chest pain, diagnostic electrocardiographic changes, and serial elevation of cardiac biomarkers as previously reported [7]. The electrocardiographic criterion for the diagnosis of STEMI was an ST-segment elevation ≥ 1 mm in >2 adjacent leads. The patients were treated according to the discretion of the senior attending physician in the CICU. Symptom duration was defined as the time of symptom onset (usually chest pain or discomfort) to the emergency room admission. Patients with a symptom duration of ≤12 h underwent primary PCI. Moreover, primary PCI was also performed on patients with a symptom duration lasting 12–24 h if the symptoms continued to persist at the time of admission. Following primary PCI, left ventricular ejection fraction was measured in all patients by bedside echocardiography, within the first 48 h of admission. The study was approved by the local institutional ethics committee. Informed consent was obtained from all patients.

The complete blood count parameters were measured using a Coulter STKS electronic counter. Blood samples for the first wr-CRP (CRP1) were drawn upon admission to the emergency department or prior to primary PCI, at the cardiac catheterization department. A second blood sample for wr-CRP (CRP2) was drawn following primary PCI, within 24 h from admission to the CICU. Quantitative wr-CRP analysis was performed by the Bayer wide-range assay, as previously described [8]. CRPv was calculated as the difference between CRP2 and CRP 1 (mg/L), divided by the time (in hours) that elapsed between the two exams. Patients were stratified into tertiles according to their CRPv. 

All patients underwent a screening echocardiographic examination within 6–72 h of CICU admission. Philips IE-33, GE, and Vivid 3 models equipped with S5-1 transducers (Philips Healthcare, Andover, MA, USA). LV diameters and interventricular septal and posterior wall width were measured from the parasternal short axis by means of a two-dimensional (2D) or a 2D-guided M-mode echocardiogram of the LV at the papillary muscle level using the parasternal short-axis view [9]. LV ejection fraction (LVEF) was calculated by the Quinones method [9]. The 16-segment model was used for scoring the severity of segmental wall motion abnormalities according to the American Society of Echocardiography [10]. Early transmitral flow velocity (E), deceleration time, and early diastolic mitral annular velocity (e’) were measured in the apical four-chamber view to provide an estimate of LV diastolic function [10]. The ratio of peak E to peak septal, lateral, and average e’ (mitral E/e’ ratio) was calculated from the average of at least three cardiac cycles. Left atrial volume was calculated using the biplane area length method at end systole [11]. Cardiac output was calculated as the product of aortic stroke volume and heart rate as demonstrated on pulse wave Doppler. Valvular regurgitation was qualitatively assessed using color Doppler according to the guidelines of the American Society of Echocardiography (normal/trivial = 1, mild = 2, moderate = 3, severe = 4) [12]. Diastolic function was assessed by integrating measurements of the mitral inflow, left atrial volume, and Doppler tissue imaging of the mitral annulus using the average annulus velocity, and classified into four categories: normal diastolic function = 0, impaired relaxation = 1, pseudo-normal = 2 and restrictive pattern = 3, based on recent guidelines [13]. Right atrial (RA) pressure was estimated by the inferior vena cava diameter as well as its response to inspiration as previously described [14]. Expiratory and inspiratory inferior vena cava (IVC) diameters and percent collapse were measured in subcostal views within 2 cm of the right atrium. IVC diameter <2.1 cm that collapsed >50% with a sniff suggested a normal RA pressure (assigned as 5 mmHg), whereas an IVC diameter >2.1 cm that collapsed <50% with a sniff suggested a high RA pressure (15 mmHg). In patients with IVC diameter <2.1 cm and no collapse (<20%) with a sniff, RA pressure was upgraded to 20 mmHg. In indeterminate cases in which the IVC diameter and collapse did not fit this paradigm, secondary indices of elevated RA pressure were integrated. If uncertainty remained, RA pressure was left as an intermediate value of 10 mmHg. In 639 patients with a measurable tricuspid regurgitation jet on Doppler echocardiography, peak systolic pulmonary artery pressure (SPAP) was estimated using the modified Bernoulli formula (4 × TRV^2^max) + RAP, where TRV max is the peak systolic tricuspid regurgitation velocity at end expiration, and RAP is the right atrial pressure.

Continuous variables were tested for normality using histograms and a Q-Q plot and compared using ANOVA testing with the post-hoc Scheffe method for differences between groups or Kruskal–Wallis one-way analysis of variance, accordingly. Normally distributed variables are expressed as a mean ± standard deviation and non-normally distributed continuous variables are expressed as median and inter quartile range (IQR). Categorical variables were expressed as percentages and compared using the Chi-square test. Binary logistic regression was employed to adjust for multiple confounders and to assess CRPv association with severe systolic dysfunction defined as EF ≤ 35% and severe diastolic dysfunction grade III. Receiver operator characteristic (ROC) curve analysis was performed to identify optimal cut-off values of CRPv for the prediction of severe systolic and diastolic dysfunction and was compared to CRP. The optimal cut-off values were calculated using the Youden’s index. All analysis with a *p*-value < 0.05 were considered statistically significant. Statistical evaluations were performed with SPSS statistics (SSPS version 25, Chicago, IL, USA). 

## 3. Results

The study cohort included a total of 1059 patients (mean age 62 ± 13 years, range 22–101, 82% males). Patients were stratified into tertiles according to their CRPv: low CRPv (0–0.27; CRPv1), intermediate CRPv (0.28–1.2; CRPv2) and high CRPv ≥ 1.2; CRPv3). Table 1 presents baselines characteristics of patients according the CRPv tertiles. Patients with a higher CRPv were older with more comorbidities and had a greater time to reperfusion.

**Figure 2:**
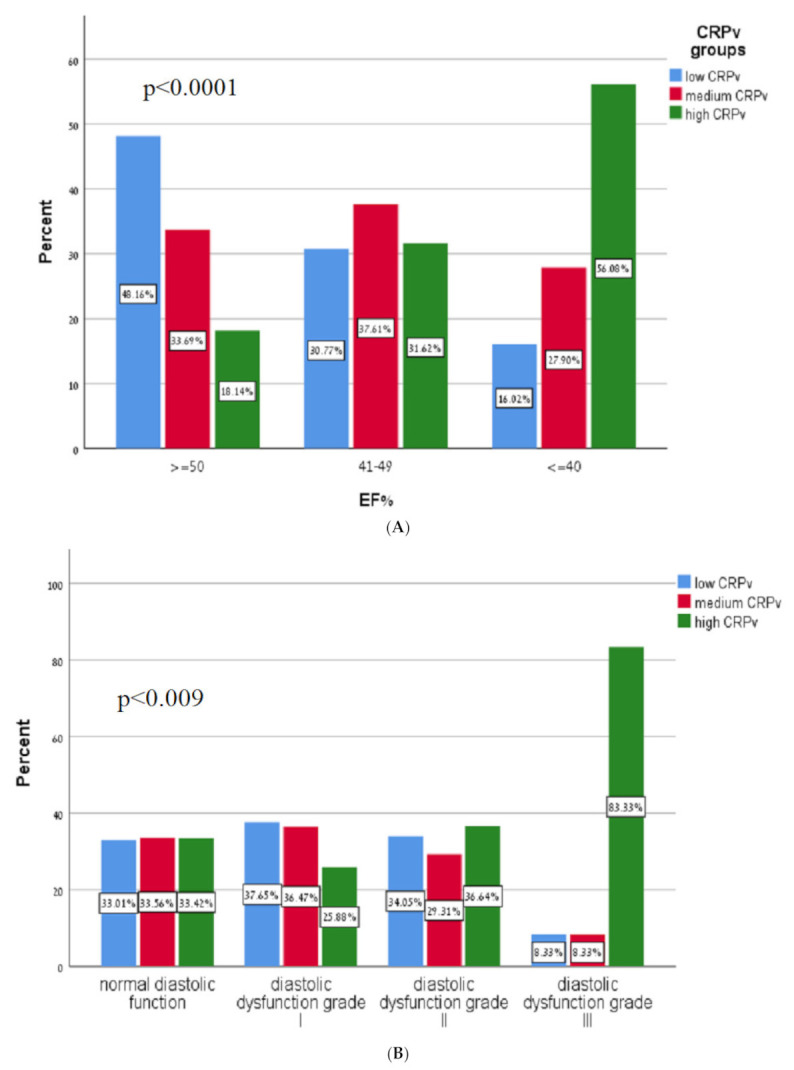
(**A**) Correlation of C-reactive protein velocity with systolic function. CRPv—C-reactive protein velocity, EF—Ejection fraction. (**B**) Diastolic function according to C-reactive protein velocity tertiles. CRPv—C-reactive protein velocity.

Table 2 presents a comparison of key echocardiographic parameters between the groups, stratified according to CRPv tertiles. We found that compared to patients in the low and medium CRPv tertiles, patients in the high CRPv tertial had a significantly lower LVEF (49% vs. 46% vs. 41%, respectively; *p* < 0.001) (Figure 2A), higher septal E/e’ (11 ± 4 vs. 11 ± 4 vs. 13 ± 6, respectively; *p* < 0.001), lateral E/e’ (9 ± 3 vs. 10 ± 4 vs. 11 ± 4, respectively; *p* < 0.001) and average E/e’ (10 ± 3 vs. 11 ± 4 vs. 12 ± 5, respectively; *p* < 0.001). Additionally, higher CRPv levels were associated with a lower deceleration time (198 ± 57 vs. 190 ± 58 vs. 175 ± 52, respectively; *p* < 0.001) and with a higher SPAP (28 ± 6 vs. 30 ± 9 vs. 34 ± 10, respectively; *p* < 0.001). Higher CRPv was also associated with an increase in diastolic dysfunction grade (*p* = 0.009) (Figure 2B).

Binary logistic regression (Table 3) found that CRPv was independently associated with both LVEF < 35% (HR 1.3 CI 95% 1.21–1.41; *p* < 0.001) and grade III diastolic dysfunction (HR 1.16 CI 95% 1.02–1.31; *p* = 0.02). 

**Figure 3:**
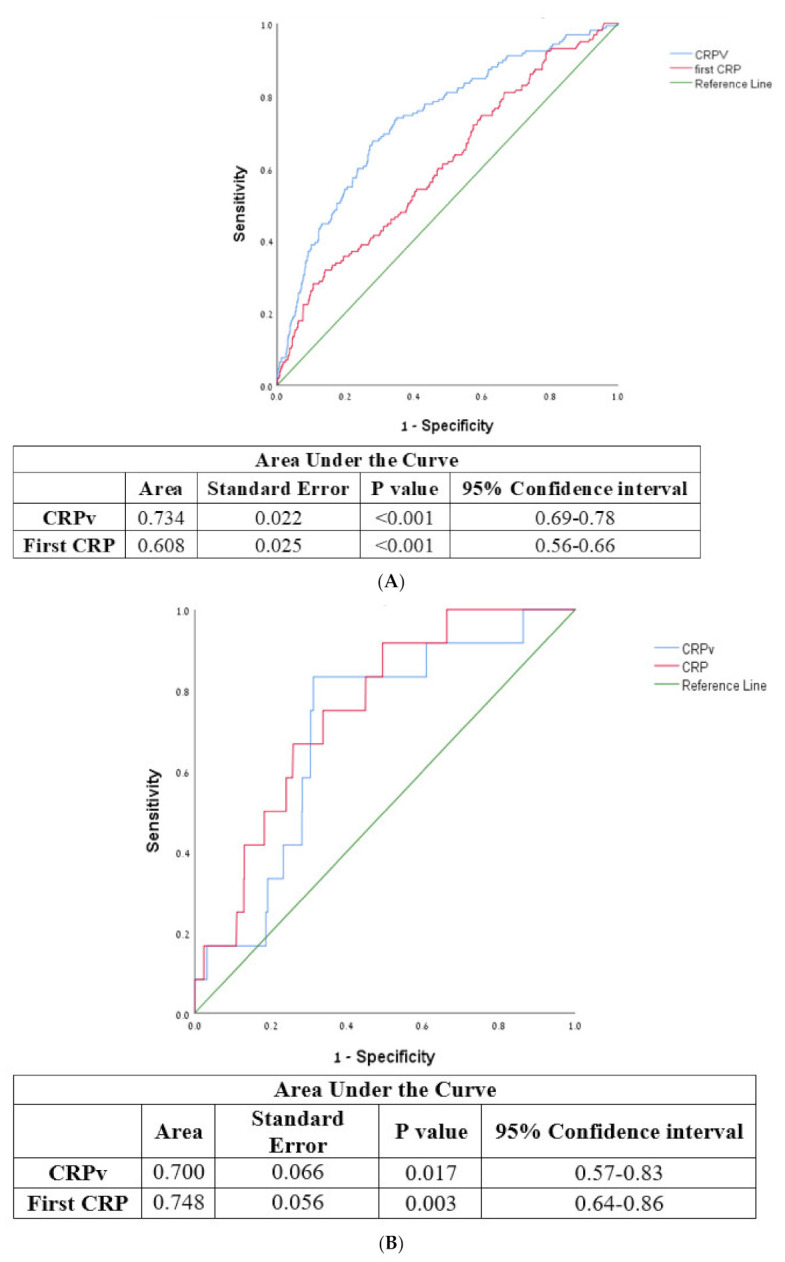
(**A**) Receiver operator characteristic curve for CRPv, CRP, and severe systolic heart failure with ejection fraction <35%. CRP—C-reactive protein, CRPv—C-reactive protein velocity. (**B**) Receiver operator characteristic curve for CRPv, CRP and severe diastolic heart failure. CRP—C-reactive protein, CRPv—C-reactive protein velocity.

According to the Receiver Operating Characteristic (ROC) curve the optimal cut-off value of CRPv for the prediction of LVEF < 35% was 1.158 with a sensitivity of 67.5% and a specificity of 71.7% (area under the curve 0.734 95% CI 0.691–0.777, *p* < 0.001). The optimal cut-off value of CRPv for the prediction of grade III diastolic failure was 1.352 with a sensitivity of 83.3% and specificity of 68.9% (area under the curve 0.7, 95% CI 0.571–0.828 *p* = 0.017). As compared to CRP, CRPv was a more sensitive and specific tool for the identification of patients at risk for developing severe systolic heart failure (Figure 3A,B). 

## 4. Discussion

The main findings of our study are that among patients presenting with STEMI treated with primary PCI, elevated CRPv was independently associated with severe systolic and diastolic dysfunction and compared to CRP, had greater sensitivity and specificity for severe systolic dysfunction. 

CRP is an acute phase protein produced in the liver in response to proinflammatory cytokines. It is one of the most studied and established biomarkers of inflammation in general, and cardiovascular inflammation in particular [15]. In patients presenting with acute MI, CRP is a powerful independent predictor for short- and long-term complications, such as in hospital death, pump failure, and left ventricular aneurysm [1,2,3,16]. Ridker et al. [17] studied the association of 11 atherothrombotic biomarkers and the development of peripheral arterial disease in healthy individuals. Among the nonlipid biomarkers, high-sensitivity CRP was the strongest predictor for the development of peripheral arterial disease. CRP has also proved to be of significant value in the evaluation of patients presenting with STEMI. A meta-analysis of seven studies comprising of approximately 700 patients with STEMI treated by primary PCI found that patients with high periprocedural CRP had a significant increase in in-hospital and follow-up, all-cause mortality, in-hospital and follow-up major adverse cardiac events, and recurrent MI. They also found that a high periprocedural CRP is an independent predictor for in-hospital target vessel revascularization [18]. 

Several studies evaluated the correlation between CRP and left ventricular function in patients with acute MI. Vanhaverbeke et al. [19] compared CRP taken at admission, peak CRP and CRP at one month in patients with acute MI and found that peak CRP correlated with left ventricular dysfunction (defined as EF ≤ 45%) at presentation and at a one-year follow up. We [1] previously reported that in patients presenting with STEMI who underwent primary PCI, elevated admission CRP was associated with echocardiographic parameters indicative of elevated filling LV pressure and worse diastolic function. 

Maximal CRP throughout hospitalization can occur at different time points and may reflect other pathophysiological processes superimposed on STEMI (e.g., sepsis, renal failure etc.) and thus may not reflect the mere response to STEMI. CRPv, focusing on CRP dynamics rather than absolute values seems to provide a greater sensitivity over CRP as an inflammatory biomarker [20]. We previously studied the correlation of CRPv to various outcomes in STEMI patients treated with primary PCI. CRPv was independently associated with higher, short-term mortality [21], and the development of acute kidney injury [22]. Interestingly, in both studies, multivariate analysis reflected CRPv superiority over CRP as a cheap and available diagnostic tool able to identify patients at risk for complications and thus guide therapy appropriately. CRPv appears to be a useful diagnostic tool that can better characterize the inflammatory dynamics during STEMI, allowing the identification of inflammation-prone patients at increased risk for a worse outcome who might benefit from anti-inflammatory intervention.

Clinical and experimental data suggests that inflammation plays a key role in pathophysiology of atherothrombotic disease [23,24,25], and several anti-inflammatory drugs have been evaluated as potential treatments in patients with cardiovascular disease. Ridker et al. [26] reported that for stable patients with prior MI and elevated CRP, compared to a placebo, treatment with Pravastatin reduced the risk of recurrent MI. Considering the lipid profile of both groups were similar, it was presumed that pravastatin efficacy is due to an anti-inflammatory mechanism and not to its lipid-lowering effect alone. Rozenbaum et al. [27] reported that STEMI patients with baseline statin therapy were more likely to have admission CRP and a second CRP within the normal range, reflecting statins anti-inflammatory effect. Canakinumab, an anti-IL-1 monoclonal antibody, was evaluated in a randomized double-blind clinical trial and showed a significant decrease in the rate of myocardial infarction and hospitalization for unstable angina, that led to urgent revascularization and coronary revascularization compared to a placebo [26]. Colchicine, a well-known anti-inflammatory drug, was also assessed in a randomized double-blind clinical trial and was found to decrease the rate of urgent hospitalization for angina, leading to revascularization and stroke compared to the placebo. However, no difference was noted in the rate of myocardial infarction and death from cardiovascular cause [28]. 

Currently, anti-inflammatory drugs are not recommended in the guidelines for patients with ACS. Nevertheless, it appears that therapy targeting inflammation will continue to be evaluated as it bears a potential and therapeutic benefit yet to be realized. 

Our study has several limitations. First, as this is a single-center retrospective study, a selection bias cannot be excluded. CRP is a nonspecific sign of ongoing inflammation. Despite excluding patients with known inflammatory/suspected infection, we were unable to determine if the rise in CRP was due to new but occult infection, whether it was secondary to inflammation during STEMI or if it was due to a different concurrent inflammatory process in the patient. Additionally, the time between the measurements of CRP1 to CRP2 ranged from as little as 2 h to 24 h, which could have led to a variation in the calculated CRP velocities of the patients. Finally, the echocardiography was performed 6–72 h following admission with no later follow up. Some of the systolic and diastolic dysfunction might be temporary due to the myocardial stunning. Current guidelines of the American Society of Echocardiography” suggests values of 8/13/18 for patients having low, intermediate, and high RA pressures. The utilization of values based on previous guideline in our cohort may have slight effect on the calculated SPAP. 

## 5. Conclusions

For STEMI patients treated with primary PCI, CRPv is a marker of both systolic and diastolic dysfunction. Further larger studies are needed to support this finding.

## Figures and Tables

**Table 1:** Baseline patient characteristics.

Variable	CRPv1 (*n* = 353)	CRPv2 (*n* = 345)	CRPv3 (*n* = 361)	*p* Value
Age (years), mean ± SD	59.35 ± 11.81	61.72 ± 13.02	64.8 ± 14.36	<0.001
Gender (Male), *n* (%)	305 (86.4%)	274 (79.7%)	289 (80.1%)	0.03
Diabetes mellitus, *n* (%)	89 (25.2%)	93 (27%)	100 (27.7%)	0.74
Hyperlipidemia, *n* (%)	191 (54.1%)	166 (48.1%)	165 (45.7%)	0.07
Current smoker, *n* (%)	199 (56.2%)	172 (48.7%)	145 (44.2%)	0.01
IHD, *n* (%)	74 (21%)	65 (18.8%)	77 (21.3%)	0.68
Family history of IHD, *n* (%)	116 (33%)	97 (28.3%)	73 (20.3%)	0.001
Atrial fibrillation, *n* (%)	8 (2.3%)	13 (3.8%)	37 (10.3)	<0.001
Hypertension, *n* (%)	150 (42.6%)	156 (45.2%)	174 (48.2%)	0.33
CKD, *n* (%)	32 (12)	40(17)	70(30)	<0.001
Peak Troponin I, ng/L, median (IQR)	4.6 (4–35)	8.9 (1.1–228)	8.2(2.2–144)	<0.001
1st CRP, mg/L, mean ± SD	6.6 ± 14.9	9.0 ± 19.2	16.4 ± 28.9	<0.001
2nd CRP, mg/L, mean ± SD	8.7 ± 1.7	24.1± 20.7	114.8 ± 72.1	<0.001
CRPv, mean ± SD	0.1 ± 0.08	0.63 ± 0.25	4.1 ± 2.82	<0.001
Time to reperfusion (minutes)—median {IQR}	175 {105–410}	194.5 {120–550}	240 {120–800}	<0.001

CKD—Chronic kidney disease, CRP—C-reactive protein, CRPv—C-reactive protein velocity, IHD—ischemic heart disease, SD—Standard deviation.

**Table 2:** Echocardiographic values stratified by CRPv tertiles.

	Low CRPv	Medium CRPv	High CRPv	*p* Value	Low CRPv and Medium CRPv	Low CRPv and High CRPv	Medium CRPv and High CRPv
Ejection Fraction (%)	49.17 ± 8.10	46.20 ± 8.35	41.90 ± 8.36	<0.001	*p* < 0.001	*p* < 0.001	*p* < 0.001
E/e’ Septal	11.04 ± 3.92	11.62 ± 4.42	13.61 ± 6.55	<0.001	*p* = 0.33	*p* < 0.001	*p* < 0.001
E/e’ Lateral	8.95 ± 3.48	9.81 ± 4.47	10.83 ± 4.76	<0.001	*p* = 0.03	*p* < 0.001	*p* = 0.01
E/e’ Average	9.87 ± 3.30	10.81 ± 4.42	12.18 ± 4.83	<0.001	*p* = 0.05	*p* < 0.001	*p* = 0.003
E (mm/sec)	72.89 ± 17.98	72.53 ± 17.86	74.59 ± 21.10	0.45	*p* = 0.98	*p* = 0.60	*p* = 0.51
A (mm/sec)	71.23 ± 19.84	74.16 ± 21.30	72.91 ± 23.65	0.33	*p* = 0.34	*p* = 0.69	*p* = 0.83
E/A	1.11 ± 0.88	1.03 ± 0.38	1.11 ± 0.49	0.14	*p* = 0.20	*p* = 0.99	*p* = 0.25
Deceleration Time (msec)	198.63 ± 57.09	190.28 ± 58.07	174.79 ± 52.62	<0.001	*p* = 0.15	*p* < 0.001	*p* = 0.002
SPAP (mmHg)	27.78 ± 6.71	30.00 ± 9.38	34.37 ± 9.80	<0.001	*p* = 0.04	*p* < 0.001	*p* < 0.001

CRP—C-reactive protein, CRPv—C-reactive protein velocity, SPAP—Systolic pulmonary artery pressure.

**Table 3:** Binary logistic regression analysis of predictors for severe systolic and diastolic heart failure.

	Severe Systolic Failure (EF ≤35%)			Severe Diastolic Failure (Grade III)		
	HR	95% CI	*p* Value	HR	95% CI	*p* Value
CRPv	1.3	1.21–1.4	<0.001	1.16	1.02–1.31	0.02
Age	1.01	0.99–1.03	0.2	1.03	0.99–1.07	0.14
Gender (Male)	0.959	0.55–1.67	0.88	0.58	0.2–1.73	0.34
Hyperlipidemia	1.14	0.72–1.8	0.586	2.1	0.87–5.09	0.1
Family history of IHD	1.01	0.6–1.71	0.995	1.31	0.51–3.38	0.58
Smoker	1.13	0.72–1.77	0.59	1.44	0.63–3.28	0.38
Hypertension	0.89	0.55–1.45	0.66	0.87	0.36–2.06	0.74
IHD	1.68	0.99–2.85	0.05	2.4	1.04–5.53	0.04
CKD	1.08	0.61–1.92	0.793	1.91	0.74–4.9	0.18
Time to reperfusion	1	1	0.71	1	0.99–1.0	0.403

CI—Confidence interval, CRPv—C-reactive protein velocity, IHD—ischemic heart disease, CKD- chronic kidney disease.

## Data Availability

Data available from the authors on request.
